# [Corrigendum] miR‑590‑5p may regulate colorectal cancer cell viability and migration by targeting PDCD4

**DOI:** 10.3892/etm.2024.12392

**Published:** 2024-01-17

**Authors:** Ting Guo, Jun Wang, Guochang Cheng, He Huang

Exp Ther Med 20:55, 2020; DOI: 10.3892/etm.2020.9183

Subsequently to the publication of the above article, an interested reader drew to the authors’ attention that a certain number of the data panels shown in Fig. 3 on p 5 for both the HCT116 and the SW480 cell line experiments appeared to contain overlapping sections of data, such that various of the panels were apparently derived from the same original sources where the results from differently performed experiments were intended to have been portrayed. After having examined their original data, the authors have realized that certain of the data panels were assembled incorrectly in this figure.

The revised version of Fig. 3, now featuring alternative data from one of the repeated experiments, is now shown on the next page. Note that these errors did not have a major impact on either the overall results or on the conclusions reported in this study. The authors regret the errors that were made during the compilation of the data in Fig. 3. All the authors agree with the publication of this corrigendum, and are grateful to the Editor of *Experimental and Therapeutic Medicine* for granting them the opportunity to publish this; furthermore, they apologize to the readership for any inconvenience caused.

## Figures and Tables

**Figure 3:**
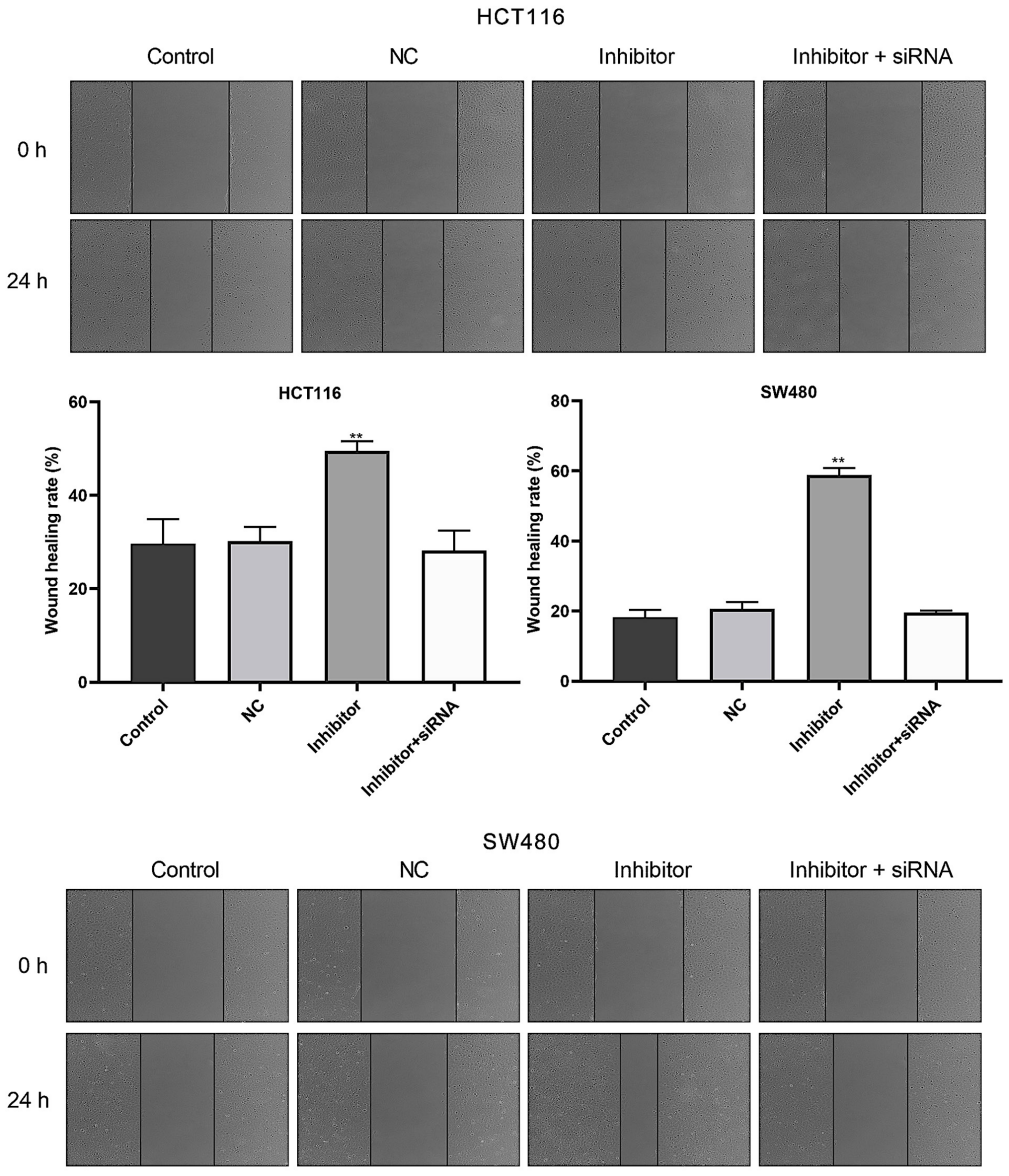
miR-590-5p inhibitor suppresses HCT116 and SW480 cell migration. The wound healing assay was conducted to examine cell migration. Magnification, x200. ^**^P<0.01 vs. control. miR-590-5p, microRNA-590-5p; NC, negative control; siRNA, small interfering RNA.

